# Phytochemical investigation, in vitro antioxidant, antibacterial activities of the leaf and fruit extracts of *Hypericum revolutum* Vahl (Amija), and essential oil composition of the leaf extract

**DOI:** 10.1186/s12906-025-05134-z

**Published:** 2025-10-22

**Authors:** Getaneh Worku Moges, Gizachew Mulugeta Manahelohe, Melesse Ababay Assege, Ayalew Temesgen Wodajo

**Affiliations:** Department of Chemistry, College of Natural and Computational Sciences, University of Gondar, P.O. Box 196, Gondar, Ethiopia

**Keywords:** Traditional Medicine, Phytochemicals, Antioxidants, *Hypericum revolutum*, Antibacterial Activity

## Abstract

*Hypericum revolutum* Vahl, a flowering plant in the *Hypericaceae* family, is traditionally used in Ethiopia to treat febrile illnesses in humans and combat diarrhea in livestock. It is known for its significant analgesic and anti-inflammatory activities. This study aims to investigate the phytochemical composition, antioxidant activity, and antibacterial efficacy of the leaf and fruit extracts, as well as the essential oil obtained from the leaves. Essential oil was extracted by hydrodistillation, while crude extracts were obtained through successive maceration using petroleum ether, chloroform, and methanol solvents. Gas chromatography-mass spectrometry (GC-MS) analysis identified major compounds, including 1R-alpha-Pinene (59.21%) and D-Limonene (7.979%). Phytochemical screening of methanol extracts revealed the presence of flavonoids, phenols, tannins, saponins, and alkaloids. Quantitative analysis revealed high total phenolic (162.04 ± 0.77 mg gallic acid equivalents (GAE)/g) and total flavonoid content (181.96 ± 8.35 mg of quercetin equivalents (QE)/g) in fruit and leaf extracts, respectively. Antioxidant activity, assessed by the DPPH assay, showed IC_50_ values of 110.07 ± 1.60 μg/mL (fruit) and 154.97 ± 4.34 μg/mL (leaf) in methanol extracts, compared to ascorbic acid (IC_50_: 27.54 ± 0.80 μg/mL). The FRAP assay revealed higher absorbance in extracts compared to ascorbic acid, indicating potent antioxidant potential. Antibacterial activity, assessed using the agar-well diffusion method, revealed significant inhibition against gram-negative (*E. coli*, *P. aeruginosa, K. pneumoniae*) and gram-positive bacteria (*S. aureus*, *S. pneumoniae*), with leaf extracts showing superior activity. The essential oil exhibited lower antibacterial efficacy. These findings support the traditional medicinal uses of *H. revolutum* and suggest its potential as a natural source of antioxidant and antibacterial agents. Further studies, including MIC/MBC determinations and advanced phytochemical analyses such as LC-MS/MS or HPLC-MS, are warranted to fully explore its therapeutic potential.

## Background

Africa is renowned for its rich biodiversity and extensive knowledge of traditional plant-based remedies for treating various illnesses, including infectious diseases. The World Health Organization (WHO) estimates that more than 80% of the population in sub-Saharan Africa uses traditional medicine made from plants for their basic healthcare needs because of its simple availability, low cost, and socio-cultural background [1]. In Ethiopia, numerous plant species are traditionally used to cure and prevent ailments in humans and animals [2, 3]. Despite facing public health challenges such as infectious diseases, chronic conditions, and limited access to modern healthcare, traditional healers and communities continue to use medicinal plants, which play a vital role not only in healthcare but also in the discovery and development of novel pharmaceuticals [3–6].

While numerous studies have focused on the phytochemistry and pharmacology of well-documented medicinal plants, there remains a significant gap in research on lesser-explored species, particularly regarding their detailed phytochemical profiles and biological activities. This gap is especially evident in the case of *Hypericum revolutum* Vahl, a medicinal plant widely used in traditional Ethiopian medicine. Despite its long history of use in treating ailments, including rheumatism, gastrointestinal disorders, and febrile illnesses, the plant’s phytochemical composition and pharmacological properties have not been sufficiently studied. Much of the existing research on the *Hypericum* genus primarily focuses on other members, leaving *H. revolutum* underexplored.

Phytochemicals in plants are generally classified into primary and secondary metabolites. Primary metabolites, such as carbohydrates, amino acids, and proteins, are essential for plant survival. In contrast, secondary metabolites, produced through pathways derived from primary metabolism, protect plants from pathogens and ultraviolet (UV) damage. These secondary metabolites include nitrogen-containing compounds (alkaloids, glucosinolates, cyanogenic glycosides), phenolic compounds (flavonoids and phenylpropanoids), and terpenes, all of which exhibit notable antiviral, antifungal, and antibiotic activities. Many of these secondary metabolites are central to the medicinal properties of plants and have been used for centuries in traditional remedies [7].

Among these, phenolic and flavonoid compounds are valuable phytochemicals with significant pharmacological, medical, and nutraceutical applications. They exert protective effects against oxidative stress and inflammation caused by reactive oxygen species (ROS). Due to their potent antibacterial, anti-inflammatory, anti-allergic, analgesic, antioxidative, and immunomodulatory properties, these compounds are highly regarded in the food and pharmaceutical industries [8, 9].

Essential oils (EOs) are complex mixtures of natural compounds, typically derived from various aromatic plant parts, such as leaves, flowers, seeds, roots, and fruits. EOs composed of up to 400 volatile compounds and have diverse and significant applications, including treating infections, acting as insect repellents and insecticides, and serving as antimalarial agents. They are also used to treat bronchitis, neurological disorders (e.g., Parkinson’s disease), ulcers, liver problems, and conjunctivitis, and to reduce stress and anxiety [10–12]. They are also has health benefits, such as antibacterial, antiviral, cardiovascular, inflammatory, immunological, hepatic, gastrointestinal, oncological, and other disease-related effects [11, 13]. Beyond their health benefits, the antioxidant activities of EOs may also enhance their ability to preserve food and feed. As a result, they hold potential as a valuable feed additive for livestock, improving the organoleptic qualities of animal products and extending their shelf life [11]. The chemical composition of essential oils can vary among the same plant species due to environmental factors. The predominant compounds found in essential oils are terpenes, bioactive molecules derived from isoprene units. By modifying these components, several forms of terpenoids can be synthesized. In addition, EOs also contain other significant families of phytochemicals, including allylphenols and propenylphenols [10].

*H. revolutum* Vahl, commonly known as Amija in Amharic, is a medicinal plant from the *Hypericaceae* family. It is a fast-growing, multistemmed evergreen shrub that can reach up to 3 meters in height, with drooping reddish-brown branches, bright yellow flowers, and reddish-brown fruit capsules. While native to sub-Saharan Africa, it has also been found in Southwest Arabia, Cameroon, the Comoro Islands, Fernando, Comoro Islands, and Indian Ocean islands [14]*.* Traditionally, it is used to treat various ailments, including rheumatism and stomach aches. In Uganda, *H. revolutum* is used to treat tuberculosis, while in Cameroon, it is employed for the treatment of protozoal diseases such as malaria [14]. The methanol extract of the aerial parts of *H. revolutum* has shown significant vasodilating effects, suggesting potential therapeutic benefits for hypertension [15, 16]. *H. revolutum* subsp. *keniense* is traditionally used in African medicine to treat diarrhea, rheumatism, nervous disorders, and wounds [12]. In Ethiopian traditional medicine, the leaves of *H. revolutum* Vahl are utilized for treating febrile illnesses in humans [4], cleaning milk utensils [17], managing diarrhea in cattle and sheep [5], and serving as healing, analgesic, and anti-inflammatory agents [18, 19]. Despite these traditional uses, H. revolutum remains underinvestigated, particularly concerning its in vitro and in vivo therapeutic potential [14].

Recent studies in Kenya have demonstrated that essential oils extracted from *H. revolutum* subsp. *keniense* leaves exhibit significant antibacterial activity and antioxidant properties [12]. These EOs showed promising levels of total phenolic content (130.46 ± 10.5 mg of gallic acid equivalent per gram) and flavonoid concentration (0.911 ± 0.04 mg of catechin equivalent per gram of dry weight), indicating their potential medicinal value [12]. However, a more comprehensive understanding of the plant’s phytochemical profile and broader pharmacological effects is needed. This study aims to fill this gap by conducting a comprehensive analysis of the phytochemical profiles, antibacterial efficacy, and antioxidant properties of *H. revolutum* fruit and leaf extracts, along with the essential oil composition of the leaf extract. The findings are expected to enhance our understanding of the plant’s medicinal potential and support its integration into modern therapeutic applications.

## Materials and methods

### Reagents and chemicals

The reagents and chemicals used in this study were ferric chloride (FeCl_3_), lead acetate (Pb(CH₃COO)₂), Wagner’s reagent (iodine in potassium iodide), aluminum chloride (AlCl_3_), hydrochloric acid (HCl), sulfuric acid (H_2_SO_4_), sodium hydroxide (NaOH), sodium dihydrogen phosphate (NaH₂PO₄), disodium hydrogen phosphate (Na₂HPO₄), sodium carbonate (Na_2_CO_3_), sodium sulfate anhydrous (Na_2_SO_4_), potassium acetate (CH_3_COOK), magnesium ribbon, Folin–Ciocalteu reagent (FCR), 2,2-diphenyl-1-picrylhydrazyl (DPPH), potassium ferricyanide (K_3_[Fe(CN)_6_]), trichloroacetic acid (CCl_3_COOH), gallic acid, quercetin, ascorbic acid, distilled water, ciprofloxacin (HiMedia Laboratories Pvt. Ltd., India), Mueller Hinton agar (Sisco Research Laboratory Pvt. Ltd., India), methanol, chloroform, petroleum ether, and dimethyl sulfoxide (DMSO). All are analytical grades.

### Test organisms

Gram-positive (*Staphylococcus aureus*; *Staphylococcus pneumoniae*) and gram-negative (*Klebsiella pneumoniae*; *Escherichia coli; Pseudomonas aeruginosa*) bacteria were used as test organisms and obtained from the Microbiology Laboratory of the Department of Biotechnology, University of Gondar, Gondar, Ethiopia. All tested organisms were clinical isolated.

### Sample collection and preparation

**Fig. 1:**
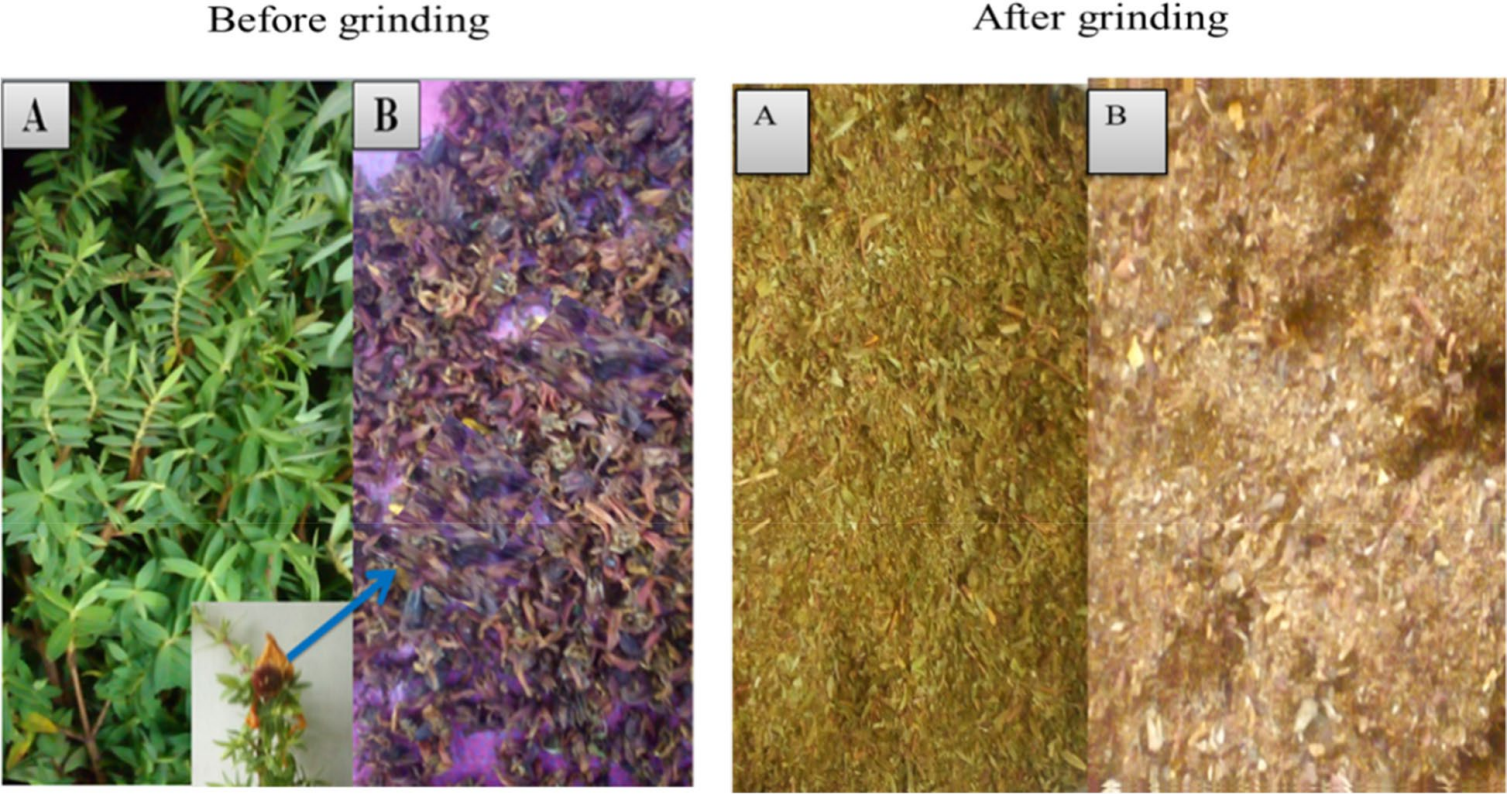
*H. revolutum* plant part of leaf (**A**) and fruit (**B**)

Fresh leaves and fruits of *H. revolutum* were collected from Simien Mountains National Park, Ethiopia, in May 2024. The plant materials were identified and authenticated by Abiyu Enyew Molla, Department of Biology, University of Gondar, Gondar, Ethiopia. A voucher specimen (GWM001/2024) was deposited at the herbarium of University of Gondar. The plant material was thoroughly washed with tap water to remove dirt and debris, followed by sterilized distilled water to minimize the risk of contamination. After washing, the materials were allowed to air dry at room temperature without direct exposure to sunlight. The dried plant materials (Fig. 1) were grounded into smaller particles using an electrical blender.

#### Maceration

**Fig. 2:**
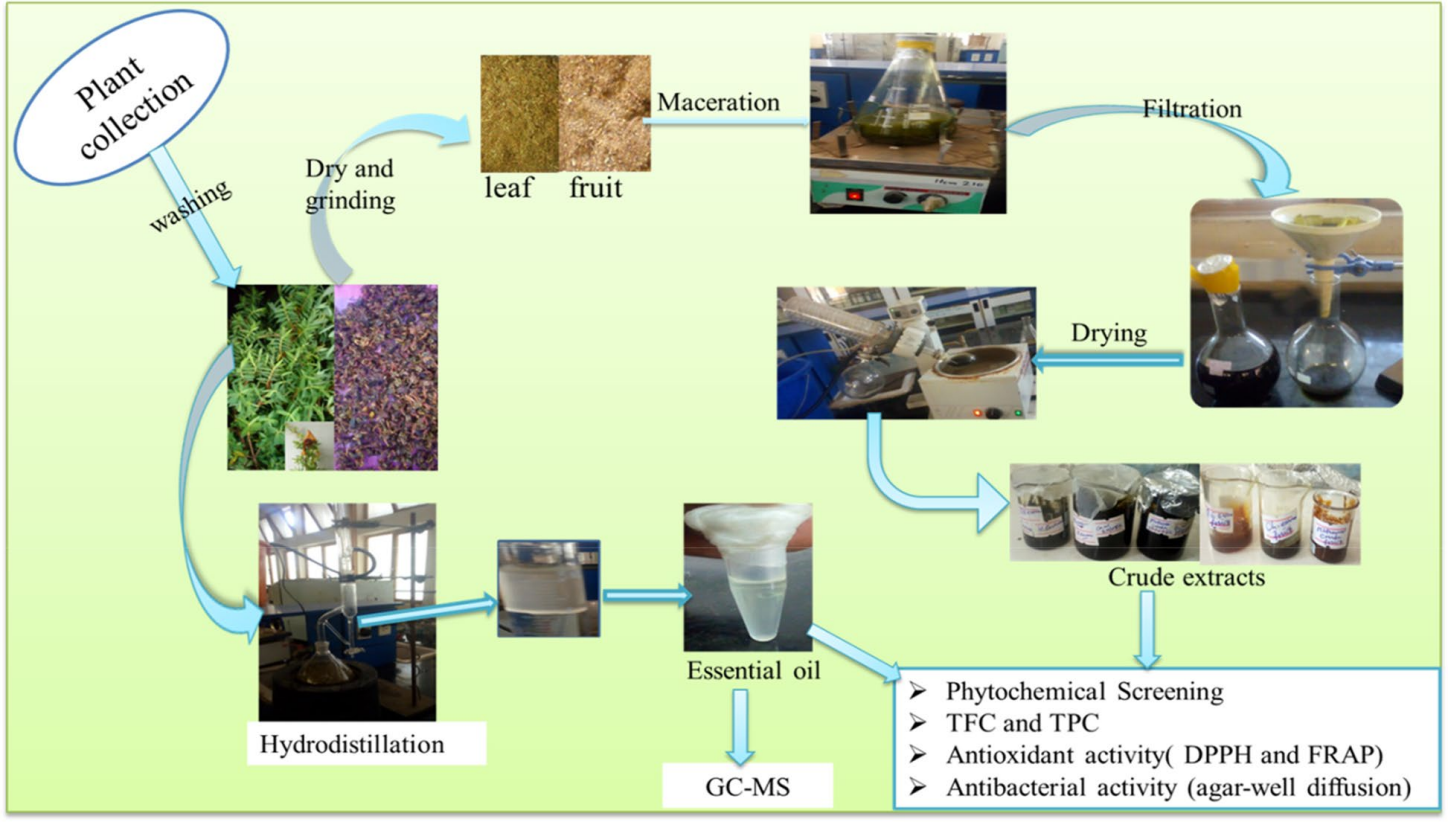
Over all methods of the experiment

The powdered plant material, consisting of 300 grams of leaves and 95 grams of fruits, was separately subjected to successive extractions using petroleum ether, chloroform, and methanol. For each plant part, 1.5 liters and 0.5 liters of each solvent were used for the leaves and fruit samples, respectively. These solvents were selected based on their varying polarities to optimize the extraction of a wide range of phytochemicals, each with distinct solubility properties. Petroleum ether, a non-polar solvent, was selected for its ability to extract non-polar compounds such as lipids, fats, and hydrocarbons, which may be poorly soluble in more polar solvents. Chloroform, a moderately polar solvent, was chosen for its versatility in extracting compounds with intermediate polarity, as it can dissolve compounds that are not fully soluble in non-polar solvents like petroleum ether but are also not highly soluble in very polar solvents like methanol. Finally, methanol, a highly polar solvent, was selected for its ability to extract a broad variety of polar bioactive compounds, such as polyphenols, flavonoids, alkaloids, and tannins, making it one of the most effective solvents for plant extraction [20]. The extractions were performed separately using maceration techniques for 72 hours with continuous agitation by an orbital shaker (Abron Exports, India), as shown in Fig. 2. The resulting solutions were filtered through Whatman No. 1 filter paper and evaporated using a rotary evaporator (Bibby RE200, UK). Finally, the extraction yield of the crude extracts was calculated and refrigerated for further analysis.

#### Hydrodistillation

The essential oil was extracted using the hydrodistillation method with a Clevenger apparatus for 3 hours, combining 130 grams of *H. revolutum* leaves with 1.5 liters of distilled water. The EO was separated using a separatory funnel, dried over anhydrous sodium sulfate, and stored in a refrigerator until analysis. Finally, the percentage yield of the EO was calculated.

### Qualitative phytochemical screening

The phytochemical screening of crude extracts was conducted to identify the main classes of compounds (alkaloids, phenols, flavonoids, tannins, saponins, glycosides, steroids, and terpenoids) present, following the standard protocol outlined by [2, 21–23].

#### Test for tannins

Approximately 0.2 g of the plant extract was taken and 1 ml of 1% lead acetate solution was added. The formation of a precipitate indicates the presence of tannins [23].

#### Test for alkaloids

Approximately 0.25 g of the crude extract was added to 5 drops of HCl and then filtered, and the filtrate was mixed with Wagner’s reagent. The formation of a brown precipitate indicated the presence of an alkaloid [22].

#### Test for saponins

Approximately 0.5 g of the extract was mixed with 5 ml of distilled water and shaken vigorously for 15 minutes. The formation of stable foam confirms the presence of saponins [21].

#### Test for flavonoids (Shinoda test)

First, 0.2 g of the plant extract was mixed with 10 ml of ethanol and filtered. Two milliliters of the filtrate were combined with a few drops of concentrated HCl and magnesium ribbon. The formation of a pink or red color confirms the presence of flavonoids [2].

#### Test for glycosides

Approximately 0.5 g of crude extract was added to 1 ml of distilled water and NaOH. The formation of a yellowish color confirmed the presence of glycosides [2].

#### Test for steroids

Approximately 0.1 g of the crude extract was combined with 10 ml of chloroform and 10 ml of sulfuric acid, and the formation of the bilayer (red top layer and greenish bottom layer) revealed the presence of steroids [2].

#### Test for terpenoids (Salkowski test)

A small amount of plant extract was dissolved in chloroform, and an equal volume of concentrated H_2_SO_4_ was added. The appearance of a reddish-brown color at the junction of two liquids indicates the presence of terpenoids [21].

#### Test for phenols

Approximately 0.25 g of the crude extract was combined with four drops of FeCl_3_ solution. The formation of a blue, green, or black coloration indicated the presence of phenols [22].

### Quantitative phytochemical analysis

#### Total phenolic content

The total phenolic content (TPC) of the extracts was determined by using the Folin-Ciocalteu colorimetric method previously described by [24], with minor modifications. Different concentrations of standard gallic acid solutions (10–200 μg/mL) were prepared. A mixture of 0.5 ml of standard, 2.5 ml of distilled water, and 0.5 ml of 10% Folin-Ciocalteu reagent (FCR) was mixed thoroughly. After 5 minutes of incubation in the dark, 1.5 ml of 7.5% Na_2_CO_3_ was added. A similar mixture was prepared using plant extracts (1 mg/mL for crude extracts and 2 mg/mL for EO in methanol) instead of a standard gallic acid solution. After 90 minutes of incubation in the dark at room temperature, the absorbance at 760 nm was measured using a microprocessor UV‒Vis double beam spectrophotometer (Abron, India). All experiments were carried out in triplicate. The TPC of the extracts was expressed as mg gallic acid equivalents (GAE) per gram of sample in dry weight using a linear equation based on the standard calibration curve.

#### Total flavonoid content

The total flavonoid content (TFC) of the extracts was determined using an aluminum chloride colorimetric assay, as previously described by [25]. To establish a calibration curve, standard quercetin solutions were prepared in methanol at concentrations ranging from 10 to 400 μg/mL. For each concentration, 0.5 mL of the quercetin solution was mixed with 1.5 mL of ethanol in a test tube. To this mixture, 0.1 mL of 10% AlCl_3_, 0.1 mL of 1 M potassium acetate, and 2.8 mL of distilled water were added. A similar mixture was prepared using plant extracts (1 mg/mL for crude extracts and 2 mg/mL for EO in methanol) instead of quercetin. After 30 minutes of incubation in the dark, the absorbance at 415 nm was measured using microprocessor UV‒Vis double beam spectrophotometer (Abron, India). All experiments were conducted in triplicate. The TFC was expressed as quercetin equivalents (mg QE/g) using a linear equation based on the standard calibration curve.

### Antioxidant activities

#### DPPH assay

The free radical scavenging activity (FRSA) was evaluated using DPPH (2, 2’-azino-bis(3-ethylbenzothiazoline-6-sulfonic acid)), following the method of [26] with minor modifications. A 0.1 mM stock solution of DPPH was freshly prepared in methanol. The DPPH solution was prepared by diluting the stock solution in a 200 mL amber volumetric flask with methanol. The absorbance values were adjusted to 1.00 ± 0.200 by adding either methanol or stock DPPH solution. Various concentrations of extracts and standard ascorbic acid (10–1000 μg/mL) were prepared. To 0.5 mL of each solution, 2.5 mL of DPPH solution was added, and the mixture was incubated at room temperature for 30 minutes in the dark. A control was prepared by combining 0.5 mL of methanol with 2.5 mL of DPPH solution. Finally, the absorbance of the solutions was measured by microprocessor UV‒Vis double beam spectrophotometer (Abron, India) at 517 nm, with methanol being used as a blank. The percentage of FRSA was calculated by using the following formula.

$$\% FRSA= \frac{AC - AO}{ AC}x100\%$$

Where % FRSA = percentage of free radical scavenging activity; AC= absorbance of the control; and AO = absorbance of the sample solution. The half maximal inhibitory concentration (IC_50_) values of the extracts and standards were determined using GraphPad Prism, with % FRSA plotted against concentration.

#### Reducing power assay/Ferric Reducing Antioxidant Power (FRAP)

Sodium phosphate buffer (0.2 M, pH 6.6): To prepare a 0.2 M sodium phosphate buffer pH 6.6, 19.2 g of sodium dihydrogen phosphate and 5.68 g of disodium hydrogen phosphate were weighed out and dissolved in 800 ml of distilled water in a 1 L volumetric flask, and the pH was adjusted to 6.6 using HCl or NaOH. Finally, the volumetric flask was filled with distilled water to the 1 L mark.

The reducing power activity was evaluated according to the method described by [27] with slight modifications. Different concentrations of crude extracts (1.0 mL) were mixed with 2.5 mL of 0.2 M sodium phosphate buffer (pH 6.6) and 2.5 mL of 1% potassium ferricyanide. The mixture was incubated at 50 °C for 20 minutes. After incubation, 2.5 mL of 10% trichloroacetic acid was added and centrifuged at 650 rpm for 10 minutes. To 2.0 mL of the upper layer, 0.4 mL of 0.1% ferric chloride and 2.0 mL of distilled water were added. The absorbance was measured at 700 nm. Higher absorbance indicates higher reducing power. The assays were carried out in triplicate, and the results are expressed as mean values ± standard deviations.

### Assessment of antibacterial activity

The antibacterial activities of extracts were tested using the agar-well diffusion method. The Mueller Hinton agar was prepared according to the manufacturer's guidelines. To prepare the agar, 39 g of powder was added to deionized water to make a final volume of one liter and mixed thoroughly. The mixture was then gently heated, brought to a boil, and autoclaved at 15 psi pressure at 121 °C for 15 minutes. Each bacterial strain used in this study was subcultured at 37 °C in Mueller-Hinton agar plates for 24 hours. The freshly prepared bacterial inoculum was evenly dispersed across the agar surface using a sterile cotton swab. Wells of approximately 6 mm in diameter were created in plates containing the solidified agar medium using a Pasteur pipette at an equal distance from each other. Fifty microliters of each extract (50 mg of crude extract or essential oil in 1 ml of dimethyl sulfoxide) were dropped in the corresponding well. The negative control of 5% dimethyl sulfoxide (DMSO) and the positive control, ciprofloxacin (5 μg/disc), were used. The plates were kept at room temperature for approximately an hour to favor diffusion and incubated at 37 °C for 24 hours. After 24 hours of incubation, the antibacterial activity was determined by measuring the diameter of the inhibition zone using a ruler [21, 22, 28].

### Characterization of the essential oil composition

The chemical composition of the essential oil isolated from the leaves of *H. revolutum* was analyzed by using gas chromatography‒mass spectrometry (GC‒MS) using the method described by [29]. GC-MS analysis was conducted on an Agilent 7820 A GC system (USA) coupled with an Agilent Technology 5977E MSD equipped with an auto-sampler (USA). The chromatographic separation was carried out on a DB-1701 micro column (30 m length × 0.25 mm internal diameter, 0.25 μm film thicknesses) at pressure of 8 psi and a flow rate 1 mL/min. Ultra-high pure helium was used as the carrier gas at constant flow mode. An Agilent G4567A auto sampler was used to inject 1.0 μL of the sample with a split less injection mode into the inlet heated to 270 °C in a total run time of 34.31 min. The oven temperature was programmed with the initial column temperature of 60 °C for 2 min. The column temperature increased to 200 °C at a rate of 10°C/min and, then heated again at rate of 3°C/min until the temperature reached 240°C. The transfer line and the ion source temperatures were 280 °C and 230 °C, respectively. The electron energy was 70 eV. Mass spectral data were collected in the range 40–650 m/z. The volatile compounds were identified by matching their mass spectra with those of reference compounds recorded in National Institute of Standards and Technology (NIST-14) mass spectral library. The relative content of each constituent was determined by area normalization [29].

### Data analysis

#### Analytical methods

The percentage yield of crude extract was calculated by using the following formula Equation (1).

1 $$\mathrm{Extraction}\;\mathrm{yield}\;(\%)=\;\;\frac{\mathrm{Mass}\;\mathrm{of}\;\mathrm{crude}\;\mathrm{extract}}{\mathrm{Mass}\;\mathrm{of}\;\mathrm{dry}\;\mathrm{plant}\;\mathrm{powder}}\;\times\;100$$

To determine the total phenolic and flavonoid contents, calibration curves of standards were constructed, leading to linear equations of the form (Equation (2).

2 $$\mathrm y=\mathrm{mx}+\mathrm b$$

where *y* represents the absorbance, *m* is the slope of the curve, *x* is the concentration of the standard, and *b* is the y-intercept.

The total phenolic content in all the samples were calculated by using the following formula Equation (3).

3 $$\mathrm{TPC}(\mathrm{mg}\;\frac{\mathrm{GAE}}{\mathrm g}\;\mathrm{dry}\;\mathrm{extract})=\;\frac{\mathrm{CV}}{\mathrm M}$$

Where *C* is the concentration of gallic acid obtained from the calibration curve in μg/mL, *V* is volume of extract in ml, and *M* is mass of extract in mg.

The total flavonoid content in all the samples was calculated by using the following formula Equation (4).

4 $$\mathrm{TFC}(\mathrm{mg}\;\frac{\mathrm{QE}}{\mathrm g}\;\mathrm{dry}\;\mathrm{extract})=\;\frac{\mathrm{CV}}{\mathrm M}$$

Where *C* is the concentration of quercetin obtained from the calibration curve in μg/mL, *V* is volume of extract in ml, and *M* is mass of extract in mg.

#### The statistical approach

The data were expressed as mean ± standard deviation, with measurements taken in triplicates. OriginPro 8 Software (version 8E) was used to generate the GC-MS chromatogram of the essential oil, while GraphPad Prism 9.3.1.471 software facilitated the determination of IC50 values, creation of calibration curves, graph construction, and calculation of the regression square coefficient (R^2^). Minitab 19.1.0.1 was also used for additional statistical analysis. One-way analysis of variance (ANOVA) followed by Tukey’s Honestly Significant Difference (HSD) test was employed to identify significant differences among means, with statistical significance set at *P* < 0.001 at 95% confidence interval.

## Results and discussion

### Percentage yields of extracts

The percentage yields of each crude extract with its respective solvent were calculated according to the equation shown below.

$$Extraction yield \left(\%\right)= \frac{Mass of crude extract}{ Mass of dry plant powder }x100$$

The yields of petroleum ether, chloroform, methanol leave extracts, and EO were 2.44, 3.298, 12.546, and 0.648%, respectively, as shown in Table 1. In the fruit extracts, the yields for petroleum ether, chloroform, and methanol extracts were 3.195, 2.527, and 14.997%, respectively, as indicated in Table 2. This study observed that the methanol extracts from the leaves and fruit of *H. revolutum* yielded the highest compared to that of petroleum ether and chloroform extracts. This could be attributed to the higher polarity of methanol, suggesting that the plant under study may contain more polar compounds.

**Table 1:** Percentage yields of *H. revolutum* leave crude extracts

**Extracts**	**Mass of extracts (g)**	**Mass of dry plant powder (g)**	**Extraction yield (%)**
Petroleum ether	7.325	300	2.44
Chloroform	9.893		3.298
Methanol	37.637		12.546
Essential oil	0.842	130	0.648

**Table 2:** Percentage yields of *H. revolutum* fruit crude extracts

**Extracts**	**Mass of extracts (g)**	**Mass of dry plant powder (g)**	**Extraction yield (%)**
Petroleum ether	3.035	95	3.195
Chloroform	2.401		2.527
Methanol	14.247		14.997

### Qualitative phytochemical screening

Phytochemicals are bioactive compounds that play a crucial role in maintaining health and act as a bridge between the food and pharmaceutical industries. These compounds are well known for their diverse pharmacological effects, including anti-inflammatory, antispasmodic, anti-allergic, antioxidant, antibacterial, antifungal, chemopreventive, neuroprotective, hypotensive, and anti-aging properties. Additionally, they can stimulate the immune system, preventing the formation of carcinogens, reducing oxidation, inhibiting cancer cell growth, reducing inflammation, inducing apoptosis, preventing DNA damage, and regulating hormones such as estrogen and insulin [30].

Blom van Staden *et al*. conducted a phytochemical screening of the aerial part of the *H. revolutum* using ethanol extract, which revealed the presence of saponins but no alkaloids, flavonoids, terpenes, phenolics, tannins, and cardiac glycosides [31]. Conversely, Andualem *et al.* reported the presence of flavonoids, tannins, and terpenoids but no alkaloids and saponins in the crude methanol extract of *H. revolutum* [32]. In this study, we performed a qualitative phytochemical screening of *H. revolutum* leaves and fruits, revealing the presence of several bioactive compounds, with variations in their distribution across different crude extracts (methanol, chloroform, and petroleum ether). The results, summarized in Table 3, showed that flavonoids, alkaloids, phenols, tannins, and saponins were detected in both leaves and fruits, with methanol proving to be the most effective solvent for extracting these compounds. Flavonoids, phenols, and saponins were found in higher concentrations in both plant parts when extracted with methanol. Alkaloids were present in leaves and fruits but at a slightly lower abundance in the methanol extract. Terpenoids were found in all three extracts of the leaves and exhibited a more diverse profile. In contrast, the fruits predominantly contained terpenoids in chloroform and petroleum ether extracts. Glycosides were consistently present in both leaves and fruits across all solvents, suggesting their widespread occurrence in *H. revolutum*. Steroids were absent in both plant parts, while tannins were primarily found in the methanol extracts of both leaves and fruits. Overall, the methanol extract proved to be the most efficient in extracting a broader range of phytochemicals, particularly flavonoids, phenols, tannins, and saponins. These findings highlight the potential of *H. revolutum* as a source of various bioactive compounds, which could be explored for traditional medicinal uses or further pharmacological research.

**Table 3:** Phytochemical screening results of *H. revolutum* leave and fruit crude extracts

**Phytochemicals**	**Plant Part**	**Crude extracts**		
		Methanol	Chloroform	Petroleum ether
Flavonoids	L	++	-	-
	F	++	-	-
Alkaloids	L	+	-	-
	F	+	-	-
Phenols	L	++	+	-
	F	++	+	-
Terpenoids	L	+	+	+
	F	-	+	+
Glycoside	L	+	+	+
	F	+	+	+
Steroids	L	-	-	-
	F	-	-	-
Tannins	L	+	-	-
	F	+	-	-
Saponins	L	++	-	-
	F	++	-	-

*L* leave, *F* Fruit, “**++**” more present “**+**” present, “**-**” absent

### Quantitative phytochemical analysis

#### Total phenolic content

The total phenolic content (TPC) of *H. revolutum* extracts has not been specifically studied. However, several studies have investigated the TPC in other *Hypericum* species. For instance, in a recent study by Afqir *et al*. measured the TPC of methanol and aqueous extracts of *H. perforatum* flowers [33]. The results showed that a higher TPC in the aqueous extract (15.26 ± 1.30 mg GAE/g) compared to the methanol extract (5.50 ± 1.13 mg GAE/g). Seyrekoglu *et al*. investigated the TPC of three *Hypericum* species from Turkey namely *H. perforatum*, *H. scabrum*, and *H. origanifolium* [34]. The results showed 128.82± 6.31, 148.31 ±4.57, and 137.03± 9.67 mg GAE/g dry weight in H. *perforatum, H. scabrum,* and *H. origanifolium*, respectively. Another study by Chimshirova *et al*. examined fresh ethanol-water (1:1) extracts of *H. perforatum* flowers and leaves, which contained phenolic content ranging from 11.0 to 56.69 mg GAE/g dry weight, while extracts from commercially sourced plants kept for nine months exhibited TPC between 9.33 and 50.98 mg GAE/g dry weight [35]. Makarova *et al*. reported a TPC range of 317.6 to 402.2 mg GAE/g (median 371 ± 49 mg GAE/g) in air-dried ethanol-water extracts of *H. perforatum* flowers, with ethanol extracts yielding values between 199.6 and 298.2 mg GAE/g (median 245 ± 26 mg GAE/g) [36]. Sengera *et al.* reported a TPC of 130.46 ± 10.5 mg GAE/g dry weight in *H. revolutum* subsp. *keniense* essential oil derived from leaves [12].

**Fig. 3:**
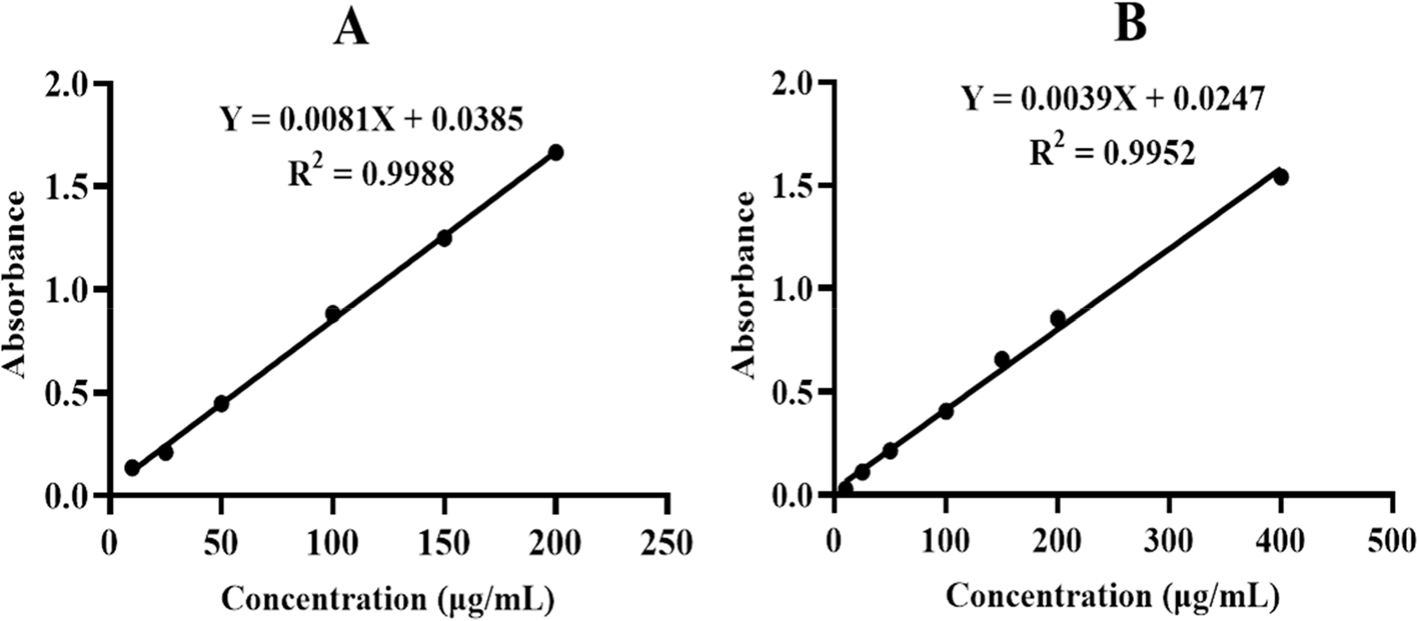
Standard calibration curve of gallic acid (**A**) and quercetin (**B**)

In the present study, the TPC of petroleum ether, chloroform, and methanol extracts from the leaves and fruits of *H. revolutum* and the essential oils derived from the leaves were quantified (Table 4). The TPC was expressed in gallic acid equivalents (GAE) using the standard curve equation (y = 0.0081x + 0.0385, R^2^ = 0.9988) shown in Fig. 3A. Notably, the methanol extracts exhibited the highest phenolic content, with values of 162.04 ± 0.77 mg GAE/g dry extract for the fruit and 130.23 ± 0.40 mg GAE/g dry extract for the leaf. In contrast, the petroleum ether extracts contained 25.29 ± 0.43 mg GAE/g dry extract in the leaf and 12.78 ± 0.54 mg GAE/g dry extract in the fruit, while the chloroform extracts had 22.65 ± 0.33 mg GAE/g dry extract in the leaf and 16.69 ± 0.19 mg GAE/g dry extract in the fruit. The essential oil derived from the leaves showed relatively lower phenolic content, with a value of 9.60 ± 0.45 mg GAE/g.

**Table 4:** TFC and TPC of the leaf and fruit extracts of *H. revolutum*

**Extracts**		**TFC (mg QE/g dry extract)**	**TPC (mg GAE/g dry extract)**
Petroleum ether	Leaf	39.31 ± 1.85^A^	25.29 ± 0.43^A^
	Fruit	35.97 ± 2.04^A^	12.78 ± 0.54^B^
Chloroform	Leaf	87.09 ± 5.34^B^	22.65 ± 0.33^C^
	Fruit	76.15 ± 1.18^B^	16.69 ± 0.19^D^
Methanol	Leaf	181.96 ± 8.35^C^	130.23 ± 0.40^E^
	Fruit	107.85 ± 1.41^D^	162.04 ± 0.77^F^
Essential oil	Leaf	4.57 ± 0.63^E^	9.60 ± 0.45^G^

Results are mean ± standard deviation (*n*=3)

Letters indicate significant differences within the same column (*P* < 0.001, α = 0.05; one-way ANOVA with Minitab 19)

Means that do not share the same letter are significantly different

The observed variation in TPC values between this study and previous reports on other *Hypericum* species can be attributed to species-specific phenolic profiles, plant part analyzed, and extraction parameters such as solvent type, extraction method, and duration [34]. Environmental factors such as climate, soil composition, altitude, and sunlight exposure influence the biosynthesis and accumulation of phenolic compounds, with plants collected from higher altitudes generally exhibiting greater phenolic content [37, 38]. The higher TPC observed in the leaves compared to fruits and in methanol extracts compared to less polar solvents reflects these influencing factors. Moreover, the elevated phenolic levels in *H. revolutum* collected from Ethiopia may be due to the region’s diverse environmental conditions, supporting its potential as a rich source of antioxidant phenolic compounds.

#### Total flavonoid content

The total flavonoid content (TFC) of *H. revolutum* extracts has not been specifically investigated in previous studies. However, researchers have examined the TFC in other species of the *Hypericum* genus. For instance, Afqir *et al*. investigated the TFC of methanol and aqueous extracts from *H. perforatum* flowers and found 10.65 ± 0.49 mg QE/g and 8.25 ± 1.08 mg QE/g, respectively [33]. Similarly, Makarova *et al*. reported the TFC of ethanol-water extracts from air-dried *H. perforatum* flowers, with values ranging from 138.4 to 175.3 mg catechin equivalents (CE)/g and a median of 160 ± 7 mg CE/g. The ethanol extract ranged from 100.4 to 167.8 mg CE/g, with a median of 122 ± 4 mg CE/g [36]. Rychlewski *et al*. determined the TFC of *H. perforatum* from both commercial and wild samples in Poland, with values ranging from 5.38 ± 0.11 to 19.96 ± 0.38 mg QE/g of dry mass [39]. Sengera *et al*. found that the flavonoid content of *H. revolutum* subsp. *keniense* essential oil derived from leaves was 0.911 ± 0.04 mg CE/g dry weight [12].

In the present study, the TFC was quantified as quercetin equivalents (QE) using the standard curve equation for quercetin (y = 0.0039x + 0.0247, R^2^ = 0.9952), as illustrated in Fig. 3B. The methanol extracts of *H. revolutum* leaves and fruits exhibited the highest TFC, with 181.96 ± 8.35 mg QE/g dry extract for the leaves and 107.85 ± 1.41 mg QE/g dry extract for the fruits. The chloroform extracts contained 87.09 ± 5.34 mg QE/g dry extract in the leaves and 76.15 ± 1.18 mg QE/g dry extract in the fruits, while the petroleum ether extracts showed 39.31 ± 1.85 mg QE/g dry extract in the leaves and 35.97 ± 2.04 mg QE/g dry extract in the fruits, as shown in Table 4. The higher efficiency of methanol in extracting flavonoids can be attributed to its polarity, which is more compatible with the polar nature of these compounds. This finding aligns with the results of [40], who observed similar trends in ethanol extracts of *Muntingia calabura* L. However, the essential oil has relatively lower flavonoid content, with a value of 4.57 ± 0.63 mg QE/g. Variations in TFC across samples may also reflect differences in plant part, solvent polarity, and geographical origin.

### Antioxidant activity

#### DPPH Assay

**Fig. 4:**
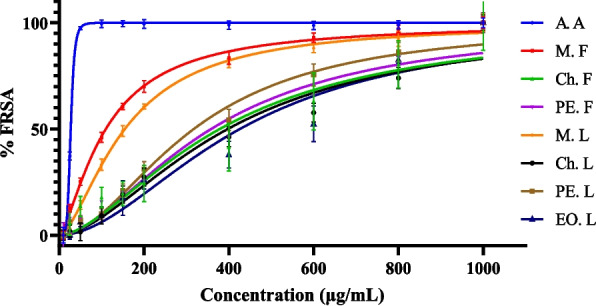
Antioxidant activity of ascorbic acid and *H.revolutum* extracts using DPPH assay

The DPPH method is one of the most remarkable methods for assessing the free radical scavenging activity (FRSA) of plant extracts. In this study, the FRSA of various solvent extracts from *H. revolutum* leaf and fruit, as well as standard ascorbic acid, are presented in Fig. 4. Among the extracts, the methanol extract has higher free radical scavenging activity than the chloroform, petroleum ether, and essential oil but lower than the standard ascorbic acid. The higher antioxidant activity of the methanol extract is due to higher TPC and TFC than the other extracts. As shown in Table 5, the methanol fruit extract of *H. revolutum* has a lower IC_50_ value of 108.5 μg/mL than the other extracts but is higher than standard ascorbic acid, which has an IC_50_ value of 27.01 μg/mL. The order of antioxidant activity of crude extract is methanol fruit (M. F) >methanol leaf (M. L) >petroleum ether leaf (PE. L) >petroleum ether fruit (PE. F)>chloroform fruit (Ch. F) >chloroform leaf (Ch. L) >essential oil leaf (EO. L). The lower IC_50_ value indicates higher free radical scavenging activity, while the higher IC_50_ value indicates weak free radical scavenging activity [41, 42].

**Table 5:** The IC_50_ value of ascorbic acid and *H.revolutum* extracts using DPPH assay (GraphPad Prism)

Extract/standard	A. A	M. F	Ch.F	PE. F	M. L	Ch. L	PE. L	EO. L
*IC*_*50*_ (μg/mL)	27.54 ± 0.80^A^	110.07 ± 1.60^B^	383.67 ± 40.73^CD^	360.4 ± 42.51^CD^	154.97 ± 4.34^B^	397.83 ± 18.16^D^	318.17 ± 19.48^C^	428.97 ± 35.49^D^
R^2^	0.9969	0.9932	0.8942	0.9558	0.9940	0.9507	0.9776	0.9259

Results are mean ± standard deviation (*n*=3), *A.A* Ascorbic acid, *M.F* Methanol fruit, *M.L* Methanol leaf, *Ch.F* Chloroform fruit, *Ch.L* Chloroform leaf, *PE.F* Petroleum ether fruit, *PE.L* Petroleum ether leaf, *EO.L* Essential oil leaf extracts

Means that do not share the same letter are significantly different (*P* < 0.001, α = 0.05; one-way ANOVA with Minitab 19)

#### Reducing power assay/Ferric Reducing Antioxidant Power (FRAP)

**Fig. 5:**
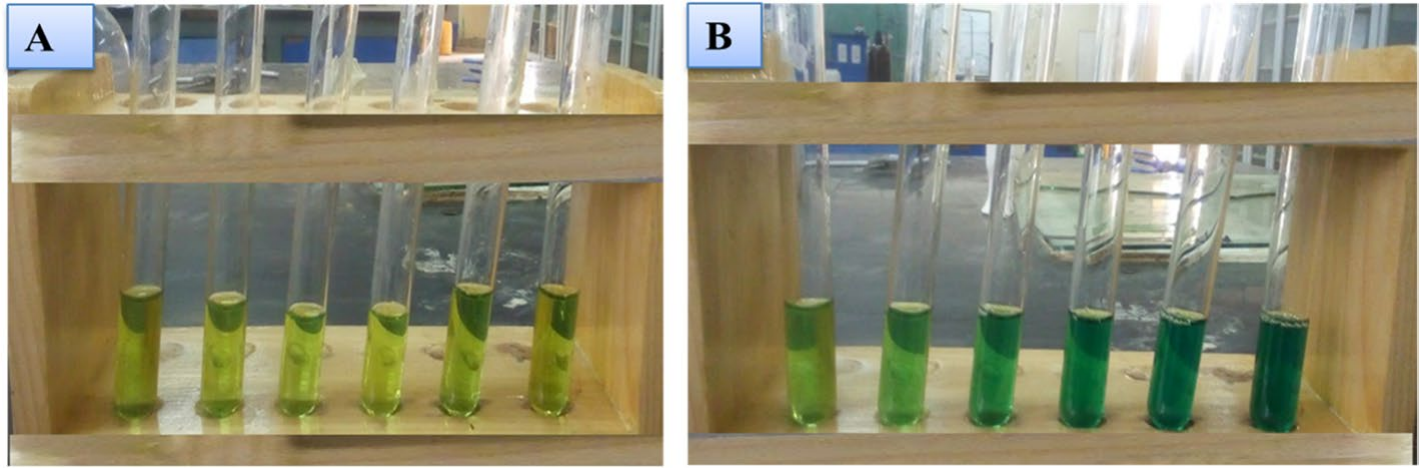
FRAP method of antioxidant activity (**A**) before addition of ferric chloride and (**B**) after addition of ferric chloride

The FRAP method is a technique used to measure the reducing power of antioxidants by monitoring the reduction of ferric iron (Fe^3^⁺) to ferrous iron (Fe^2^⁺) without the involvement of free radicals [12]. In recent FRAP experiments, potassium ferricyanide is commonly used as the primary ferric reagent, with the reduction process monitored spectrophotometrically. When Fe^3^⁺ is added to the solution, the color changes from yellow to Prussian blue, indicating a higher reducing power of the plant extract, as shown in Fig. 5. The formation of Prussian blue may result from two possible processes. Antioxidants in the plant extract can convert ferricyanide to ferrocyanide, which binds the Fe^3^⁺ ions and forms Prussian blue, or they can directly reduce Fe^3^⁺ to Fe^2^⁺ [43].

**Fig. 6:**
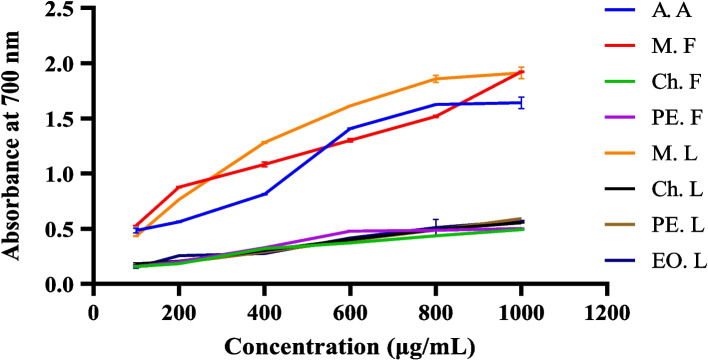
Antioxidant activity of *H. revolutum* leaf and fruit extracts by FRAP assay

In this study, the ferric-reducing antioxidant power of different solvent extracts of *H. revolutum* was measured at various concentrations from 100 to 1000 μg/mL. The result showed that the absorbance increases as the concentration increases. High antioxidant activity was observed by an increase in absorbance [12]. Among the tested extracts, at concentrations of 100, 200, and 1000 μg/mL, the methanol extract of fruit had the maximum absorbance compared with ascorbic acid and other extracts, as shown in Fig. 6 and Table 6. On the other hand, at concentrations of 400, 600, and 800 μg/mL, the methanol extracts of the leaf had higher absorbance. This study revealed that the FRAP of *H. revolutum* extracts was dose-dependent on antioxidant capacity. However, the petroleum ether, chloroform extracts, and essential oils had lower FRAP than methanol extracts and ascorbic acid.

**Table 6:** FRAP of *H. revolutum* leaf and fruit extracts

**Concentration (μg/mL)**	**Absorbance at 700 nm**							
	M. F	M. L	Ch. L	Ch. F	PE. L	PE. F	EO. L	A. A
100	0.528 ± 0.002^a^_A_	0.431 ± 0.002^a^_B_	0.181 ± 0.008^a^_B_	0.157± 0.007^a^_CD_	0.155 ± 0.003^a^_CD_	0.160 ± 0.009^a^_CD_	0.149 ± 0.007^a^_D_	0.483 ± 0.022^a^_E_
200	0.878 ± 0.006^b^_A_	0.763 ± 0.002^b^_B_	0.206 ± 0.001^b^_C_	0.183 ± 0.001^b^_D_	0.191 ± 0.003^b^_DE_	0.200 ± 0.004^b^_CE_	0.256 ± 0.001^b^_F_	0.563 ± 0.007^b^_G_
400	1.083 ± 0.023^c^_A_	1.281 ± 0.008^c^_B_	0.303± 0.001^c^_CD_	0.320 ± 0.003^c^_C_	0.290 ± 0.001^c^_DE_	0.327 ± 0.004^c^_C_	0.276 ± 0.003^b^_E_	0.812 ± 0.005^c^_F_
600	1.300 ± 0.016^d^_A_	1.613 ± 0.003^d^_B_	0.401± 0.002^d^_CD_	0.372 ± 0.005^d^_E_	0.394 ± 0.003^d^_D_	0.478 ± 0.007^d^_F_	0.416 ± 0.002^c^_C_	1.408 ± 0.005^d^_G_
800	1.517 ±0.012^e^_A_	1.857 ± 0.033^e^_B_	0.493 ± 0.002^e^_C_	0.436 ± 0.002^e^_C_	0.487 ± 0.002^e^_C_	0.486 ± 0.001^ef^_C_	0.510 ± 0.075^d^_C_	1.626 ± 0.004^e^_D_
1000	1.923 ±0.007^f^_A_	1.913 ± 0.053^e^_A_	0.554 ± 0.002^f^_BCD_	0.493 ± 0.006^f^_C_	0.591 ± 0.002^f^_B_	0.501 ± 0.005^f^_CD_	0.571± 0.003^d^_BD_	1.642 ± 0.053^f^_E_

All result is as mean ± standard deviation (*n*=3)

The letters indicate significant differences within the same column (superscript lowercase letters) and the same row (subscript uppercase letters) (*P* < 0.001, α = 0.05; one-way ANOVA with Minitab 19)

Means that do not share the same letter are significantly different

### Antibacterial activity

In the previous study, Keskes *et al*. investigated the antibacterial activity of 80% methanol crude extract of *H. revolutum* leaves. The study reported an inhibition zone of 13.3 and 5.6 mm against *E. coli* and *S. typhi*, respectively. However, the extract was ineffective against *S. typhimurium* and *S. paratyphi* [19]. Similarly, Andualem *et al*. investigated the antibacterial activity of the methanol extract of *H. revolutum* against *S. paratyphi, S. typhimurium, S. typhi*, *Shigella*spp., *S. aureus*, *P. aeruginosa*, and *E. coli*. The growth inhibition zones were 12, 10, and 9 mm against *P. aeruginosa*, *E. coli*, and *Shigella* spp., respectively, while inactive against S*. paratyphi, S. typhimurium, S. typhi*, and *S. aureus* [32].

**Fig. 7:**
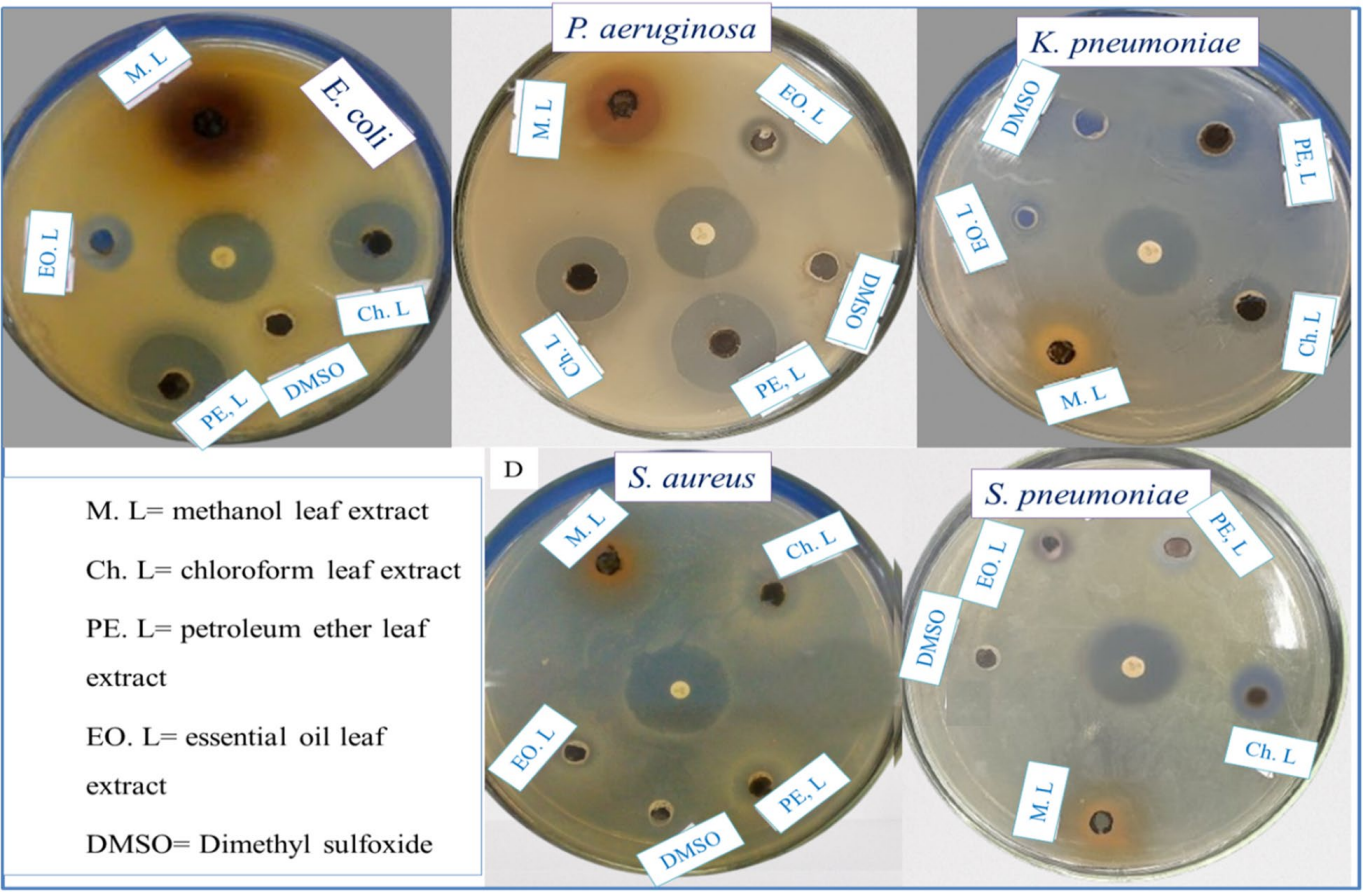
The antibacterial activity of *H. revolutum* leaf extracts and essential oil

In this study, the antibacterial activity of the crude leaf extracts and essential of *H. revolutum* was tested against five clinically isolated bacterial strains, as shown in Table 7 and Fig. 7. One-way ANOVA with Tukey’s HSD test confirmed statistically significant differences in inhibition zones (*p* < 0.001). The crude petroleum ether extract displayed the highest antibacterial activity against *P. aeruginosa* and *E. coli*, with an inhibition zone of 20.67 ± 0.58 mm and 20.33 ± 0.58 mm, respectively. On the other hand, the methanol crude extract displayed the highest antibacterial activity against *E coli*, *K. pneumoniae*, and *P. aeruginosa* with an inhibition zone of 20.00 ± 1.00 mm, 18.67 ± 1.53 mm, and 18.00 ± 1.00 mm, respectively. As a comparison, the standard drug ciprofloxacin exhibited an inhibition zone of 21.67 ± 1.53 mm, 21.333 ± 0.58 mm, 21.00 ± 1.00 mm, 19.67 ± 1.16 mm, and 19.33 ± 1.53 against *E. coli*, *P. aeruginosa*, *K. pneumoniae*, *S. aureus*, and *S. pneumoniae*, respectively. In addition, the chloroform extract displays an inhibition zone of 18.33 ± 0.58 mm against *E. coli*, 18.00 ± 1.00 mm against *P. aeruginosa*, and 10.33 ± 0.58 mm against *K. pneumoniae*, *S. aureus*, and *S. pneumoniae*. The petroleum ether crude has displayed the highest inhibition zone of 20.67 ± 0.58 and 20.33 ± 0.58 against *P. aeruginosa* and *E. coli*, respectively. The essential oil has the lowest antibacterial activity than the other three organic solvent extracts, with an inhibition zone of 7.67 ± 1.15 mm, 7.33 ± 0.58 mm, 3.33 ± 0.58 mm, and 2.00 ± 1.00 mm against *E. coli*, *S. pneumoniae*, *P. aeruginosa*, and *K. pneumoniae*, respectively, but it is inactive against *S. aureus*. The negative control (5% DMSO) showed no inhibition, confirming that the observed antibacterial effects were due to the plant extracts.

**Table 7:** Antibacterial test results of *H. revolutum* leave crude extracts and its essential oil

Test organism	Zone of inhibition (mm) at 50 mg/mL crude extract				
	Methanol	Chloroform	Petroleum ether	Essential oil	Ciprofloxacin
*E. coli*	20.00 ± 1.00^a^_AB_	18.67 ± 0.58^a^_B_	20.33 ± 0.58^a^_AB_	7.67 ± 1.15^a^_C_	21.67 ± 1.53^a^_A_
*P. aeruginosa*	18.00 ± 1.00^a^_A_	18.00 ± 1.00^a^_A_	20.67 ± 0.58^a^_B_	3.33 ± 0.58^b^_C_	21.33 ± 0.58^a^_B_
*K. pneumoniae*	18.67 ± 1.53^ab^_A_	10.33 ± 0.58^b^_B_	9.33 ± 0.58^b^_B_	2.00 ± 1.00^b^_C_	21.00 ± 1.00^a^_A_
*S. aureus*	15.00 ± 1.00^bc^_A_	10.33 ± 0.58^b^_B_	9.67 ± 0.58^b^_B_	-	19.67 ± 1.16^a^_C_
*S. pneumoniae*	14.67 ± 1.16^c^_A_	10.33 ± 0.58^b^_B_	11.67 ± 1.16^c^_B_	7.33 ± 0.58^a^_C_	19.33 ± 1.53^a^_D_

All results are indicated as mean ± standard deviation (*n*=3)

Means with different superscript lower-case alphabets within the same column are significantly different, whereas means with different subscript uppercase alphabets within the same row are significantly different (*P* < 0.001, α = 0.05; one-way ANOVA with Minitab 19)

The zone of inhibition of negative control is zero millimeters

The relatively weak antibacterial activity of the essential oil may be attributed to the volatility of its constituents and poor solubility in the aqueous agar medium. Despite being dissolved in DMSO, the absence of an emulsifying agent likely limited its dispersion and interaction with bacterial cells. Additionally, essential oil components may have evaporated during incubation, reducing their bioavailability and overall efficacy [44]. These factors may have contributed to the lowest antibacterial effects observed in the present study and should be considered when interpreting in vitro results for volatile plant-derived oils.

**Fig. 8:**
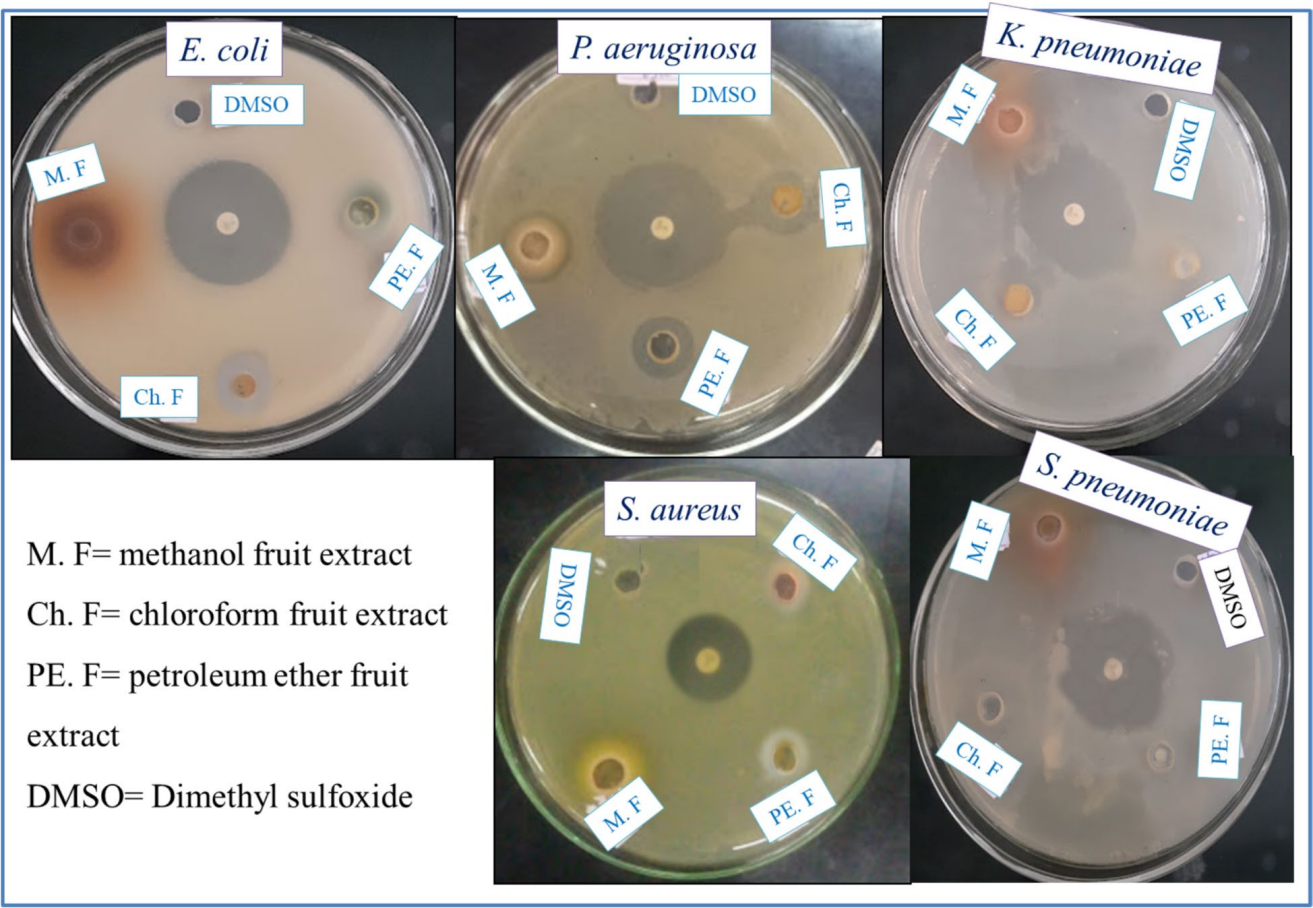
The antibacterial activity of *H. revolutum* fruit extracts

The fruit crude extracts of *H. revolutum were* also tested for their antibacterial activity against five bacterial strains, as shown in Table 8 and Fig. 8. One-way ANOVA followed by Tukey’s HSD test revealed significant differences in inhibition zones between the plant extracts and the standard antibiotic ciprofloxacin (*p* < 0.001). Results showed that the crude methanol extract exhibited the highest antibacterial activity compared with the petroleum ether and chloroform extracts. However, its effectiveness was lower than that of the standard drug ciprofloxacin. Specifically, the inhibition zones of the methanol extract of *H. revolutum* fruit were 15.00 ± 1.00, 15.67 ± 1.53, 12.67 ± 1.53, 13.67 ± 2.08, 14.68 ± 1.53 mm against *E. coli, P. aeruginosa, K. pneumoniae, S. aureus*, *and S. pneumoniae*. In contrast, the standard ciprofloxacin has an inhibition zone of 22.00 ± 1.00, 21.33 ± 1.16, 20.33 ± 0.58, 19.33 ± 0.58, and 19.00 ± 1.00 mm against *E. coli*, *P. aeruginosa*, *K. pneumoniae*, *S. aureus*, and S*. pneumoniae*, respectively. The antibacterial activity of the organic extracts of fruit *H. revolutum* is in the order of methanol >chloroform >petroleum ether extracts. The more polar organic solvent of methanol extract has higher antibacterial activity than the least polar organic solvents; this result agrees with the study by [45], who reported the methanol extract of *H. perforatum* has higher antibacterial activity than the chloroform, ethyl acetate, and n-hexane extracts.

**Table 8:** Antibacterial test results of *H. revolutum* fruit crude extracts

Test organism	Zone of inhibition (mm) at 50 mg/mL crude extract			
	Methanol	Chloroform	Petroleum ether	Ciprofloxacin
*E. coli*	15.00 ±1.00^a^_A_	8.67 ± 0.577^a^_B_	7.00 ± 1.00^a^_B_	22.00 ±1.00^a^_C_
*P. aeruginosa*	15.67 ± 1.53^a^_A_	12.33 ± 0.58^b^_AB_	10.67 ± 0.58^b^_B_	21.33 ± 1.16^ab^_C_
*K. pneumoniae*	12.67 ± 1.53^a^_A_	7.33 ± 0.58^ac^_B_	7.00 ± 1.73^a^_B_	20.33 ± 0.58^ab^_C_
*S. aureus*	13.67 ± 2.08^a^_A_	6.33 ± 0.58^c^_B_	6.33 ± 1.53^a^_B_	19.33 ± 0.58^b^_C_
*S. pneumoniae*	14.68 ± 1.53^a^_A_	6.67 ± 0.58^c^_B_	8.33 ± 1.16^ab^_B_	19.00 ± 1.00^b^_C_

All results are written as mean ± standard deviation (*n*=3)

Means with different superscript lower-case alphabets within the same column are significantly different, whereas means with different subscript uppercase alphabets within the same row are significantly different (*P* < 0.001, α = 0.05; one-way ANOVA with Minitab 19)

The zone of inhibition of negative control is zero millimeters

Despite the promising antibacterial effects, this study has some limitations that should be addressed in future research. First, while the agar-well diffusion method provided qualitative data, determining the Minimum Inhibitory Concentration (MIC) and Minimum Bactericidal Concentration (MBC) is essential for quantifying the concentration-dependent effects and establishing the therapeutic potential of the extracts. Incorporating MIC and MBC data would also enable a more accurate comparison with ciprofloxacin. Additionally, the study focused on only five bacterial strains. Future research should expand the bacterial panel to include multidrug-resistant pathogens for a broader evaluation of *H. revolutum's* antibacterial potential.

Overall, the extracts showed greater antibacterial activity against Gram-negative bacteria (*E. coli, P. aeruginosa, K. pneumoniae*) compared to Gram-positive strains (*S. aureus, S. pneumoniae*. This trend likely reflects structural differences in bacterial cell walls. Gram-negative bacteria possess an outer membrane rich in lipopolysaccharides, which can act as a barrier to hydrophilic compounds but may be more vulnerable to lipophilic ones that disrupt membrane integrity. Moreover, plant-derived flavonoids and other secondary metabolites often demonstrate greater efficacy against Gram-negative bacteria when their lipophilicity (e.g., LogP values) falls within an optimal range [46, 47]. These findings suggest that the antibacterial activity of *H. revolutum* extracts is influenced by the physicochemical properties of their phytochemicals, with a greater effect on Gram-negative pathogens, although some activity against Gram-positive strains supports a degree of broad-spectrum potential.

### Essential oil composition

Essential oils contain valuable secondary metabolites derived from various plant species. They are useful for creating herbal medicines and can function as preservatives and antioxidants in pharmaceuticals, agriculture, food, and cosmetics. Additionally, due to their diverse active compounds, these substances exhibit antiviral, antifungal, antibacterial, insecticidal, and allelopathic properties. These qualities can enhance product quality, extend shelf life, and protect plants from pests and diseases [48].

The essential oil composition of *H. revolutum* has not been reported previously in Ethiopia. However, a study by Sengera *et al*. in Kenya examined the essential oil composition of the *H. revolutum* subspecies *kinase* flower and leaf. They reported that the major constituents are caryophyllene (22.1%), α-farnesene (9.2%), 1-octyl trifuoroacetate (7%), 2-ethyl-2-methyl-oxirane (6.3 %), (Z, E)−3, 7, 11-trimethyl-1, 3, 6, 10-dodecatetraene (4.5%), α-copaene (4.3%)%), 3-(2-bromoethyl) dihydro-2 (3H)-furanone (3.1 %), α-acetobutyrolactone (2.8 %), α.–pinene (2.3 %), trans-1,2-bis- (1-methylethenyl) cyclobutane (2.3 %), and cis-calamenene (2.2%) in the leaf. In the same study, 7- (1-methylethylidene) bicyclo (4.1.0) heptane (15 %), (E)- 2,7-dimethyl-3-octen-5-yne (11 %). 2, 3,4-trimethylhexane (10.5 %), 2-ethyl-2-methyl-oxirane (8.8 %), caryophyllene (8.5 %), 1-octyl trifuoroacetate (7.9 %), 4,4-dimethyloxazolidine (6.6 %), D-camphene (5.3 %), and α-copaene (3.2 %) as a major constituents of the flower [12]. On the other hand, several studies have been conducted on the essential oil composition of the genus *Hypericum* in different countries. Semerdjieva *et al*. studied the essential oil composition of seven Bulgarian *hypericum* species and reported that the major components are α-pinene (6.2–23.86.2.86 %), β-pinene (2.28–29.37.28.37 %), β-caryophyllene (3.85–16.08.85.08%), D-limonene (3.75–14.44.75.44%), caryophyllene oxide (1.97–15.90.97.90%), and alpha.-Muurolene (3.13%) [49]. Another report by Grafakou *et al*. in 2022 found that α-pinene was identified as a major component in *hypericum* species, with concentrations of up to 88.3% [50].

**Fig. 9:**
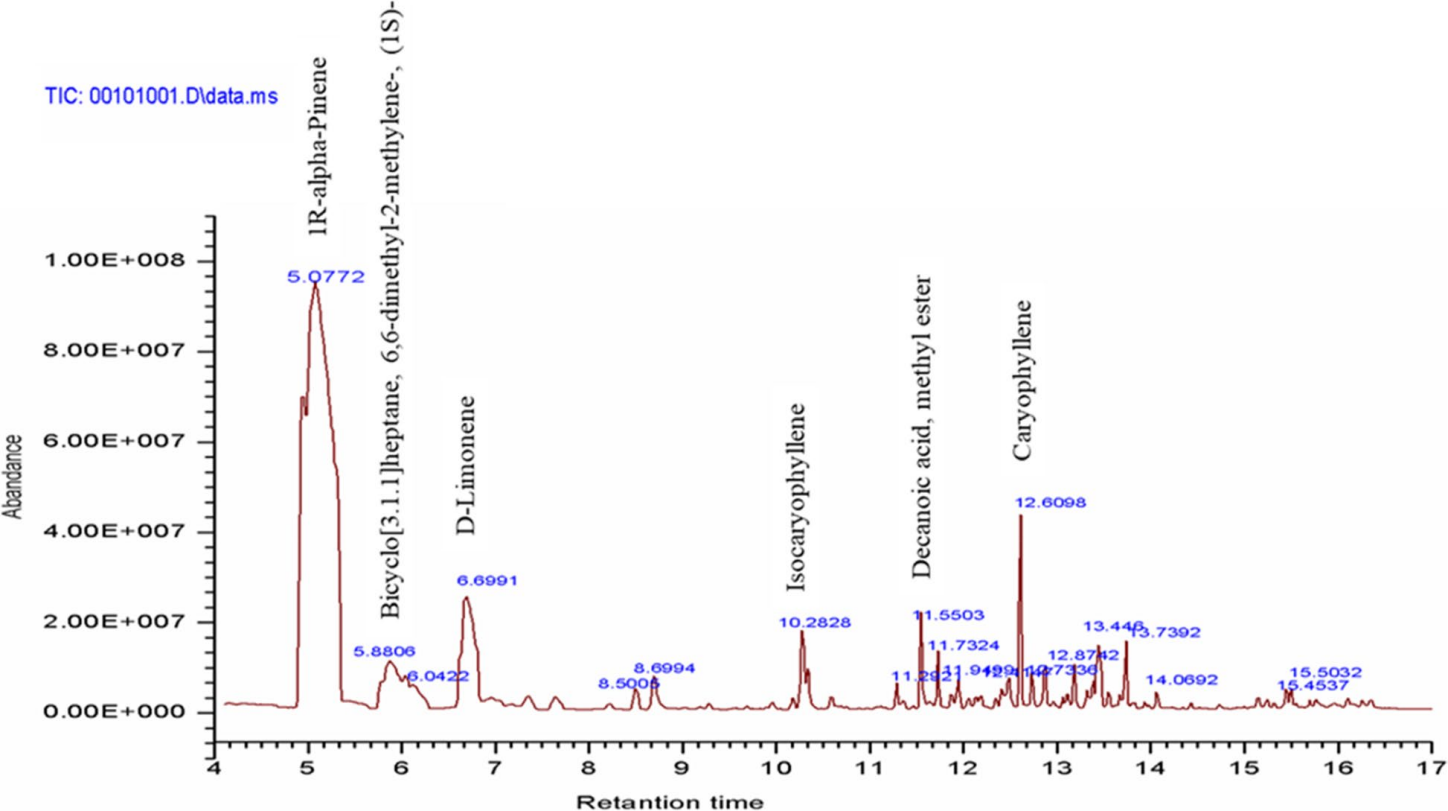
GC- MS chromatogram of *H. revolutum* leaf essential oil

In the present study, 23 compounds were identified using GC-MS from the essential oils of *H. revolutum* leaves, as shown in Fig. 9. The primary components of the essential oils were 1R-alpha-Pinene (59.21%), D-limonene (7.979%), bicyclo[3.1.1]heptane, 6,6-dimethyl-2-methylene-, (1S)- (3.1481%), isocaryophyllene (2.6548%), caryophyllene (2.6362%), and 1,2,4-Metheno-1H-indene, octahydro-1,7a-dimethyl-5-(1-methyl ethyl)-, [1S-(1.alpha.,2.alpha.,3a.beta.,4.alpha.,5.alpha.,7a.beta.,8S*)]- (2.1322%), as detailed in Table 9. Additionally, our analysis revealed the presence of naphthalene derivatives in the essential oils, identified as a minor component with concentrations ranging from 0.307% to 1.1435%. This finding is consistent with the findings reported by [51], which documented the occurrence of naphthalene derivatives in the leaves of *H. triquetrifolium* from Iraq.

**Table 9:** GC-MS profile of the leave essential oil composition of *H. revolutum*

**Peak**	**Retention time**	**Compound**	**CAS**	**% content of total**	**Qual**
1	4.9485	1R-alpha-Pinene	007785-70-8	10.8453	89
2	5.0772	1R-alpha-Pinene	000080-56-8	59.2069	90
3	5.8806	Bicyclo[3.1.1]heptane, 6,6-dimethyl-2-methylene-, (1S)-	018172-67-3	3.1481	97
4	6.0422	.beta.-Pinene	000127-91-3	0.3959	97
5	6.6991	D-limonene	005989-27-5	7.979	98
6	8.5005	2,4,6-Octatriene, 2,6-dimethyl-, (E,Z)-	007216-56-0	0.6246	97
7	8.6994	Nonanoic acid, methyl ester	000110-42-9	1.1555	91
8	10.2828	Isocaryophyllene	000087-44-5	2.6548	99
9	11.2921	1,5,5-Trimethyl-6-methylene-cyclohexene	000514-95-4	0.3953	93
10	11.5503	Decanoic acid, methyl ester	000110-42-9	1.4855	98
11	11.7324	Copaene	003856-25-5	0.9505	99
12	11.9499	Tricyclo[2.2.1.0(2,6)]heptane, 1,7-dimethyl-7-(4-methyl-3-pentenyl)-, (-)-	000512-61-8	0.5775	95
13	12.4147	alpha.-Muurolene	010208-80-7	0.4136	96
14	12.4864	Benzene, 1-(1,5-dimethyl-4-hexenyl)−4-methyl-	000644-30-4	0.7449	98
15	12.6098	Caryophyllene	000087-44-5	2.6362	99
16	12.7336	Naphthalene, 1,2,3,4,4a,5,6,8a-octahydro-7-methyl-4-methylene-1-(1-methylethyl)-, (1.alpha.,4a.beta.,8a.alpha.)-	039029-41-9	0.6331	97
17	12.8742	Naphthalene, 1,2,3,5,6,8a-hexahydro-4,7-dimethyl-1-(1-methylethyl)-, (1S-cis)-	000483-76-1	0.8538	98
18	13.1888	gamma.-Muurolene	030021-74-0	0.6741	98
19	13.3243	Naphthalene, 1,2,3,4-tetrahydro-1,6-dimethyl-4-(1-methylethyl)-, (1S-cis)-	000483-77-2	0.309	95
20	13.446	1,2,4-Metheno-1H-indene, octahydro-1,7a-dimethyl-5-(1-methylethyl)-, [1S-(1.alpha.,2.alpha.,3a.beta.,4.alpha.,5.alpha.,7a.beta.,8S*)]-	022469-52-9	2.1322	83
21	13.7392	Naphthalene, 1,2,4a,5,8,8a-hexahydro-4,7-dimethyl-1-(1-methylethyl)-, [1S-(1?,4a?,8a?)]-	000483-76-1	1.1435	98
22	14.0692	4-isopropyl-1,6-dimethyl-1,2,3,4-tetrahydronaphthalene	1000378-99-6	0.3072	95
23	15.4537	Aromandendrene	000489-39-4	0.3354	91
24	15.5032	Isoledene	095910-36-4	0.3982	91

## Conclusion

In conclusion, *H. revolutum* demonstrates considerable potential as a source of bioactive phytochemicals with significant antioxidant and antibacterial properties. Methanol extracts of both leaf and fruit revealed diverse secondary metabolites, including flavonoids, phenols, tannins, saponins, and alkaloids, and exhibited the highest levels of total phenolic and flavonoid content. These extracts also showed significant antioxidant activity and antibacterial effects. In contrast, chloroform and petroleum ether extracts displayed lower phytochemical profiles. Notably, the leaf extracts exhibited greater antibacterial activity than fruit extracts against the tested bacterial strains. The essential oil obtained from the leaves contains many volatile compounds, with 1R-alpha-Pinene identified as the major constituent.

This study expands current knowledge by providing a phytochemical profile and biological activity of *H. revolutum*, a species that has received limited scientific attention compared to other members of the *Hypericum* genus. The findings support the therapeutic potential of its methanol extracts, particularly from the leaves, for future development as natural antibacterial and antioxidant agents.

However, this study has several limitations that should be addressed in future research. The use of the agar-well diffusion method for antibacterial assessment does not provide precise quantitative data, as MIC and MBC values were not determined. Furthermore, the phytochemical analysis was limited to preliminary screening, without advanced techniques such as LC-MS/MS or HPLC-MS, which are essential for accurate identification and quantification of bioactive compounds. To fully elucidate the therapeutic potential of *H. revolutum*, future studies should incorporate MIC and MBC assays and employ advanced analytical methods to characterize the active constituents responsible for the observed antioxidant and antibacterial activity.

## Data Availability

The data used to support the findings of this study are included within the article.
